# Immunomodulatory Activity and Its Mechanisms of Two Polysaccharides from *Poria cocos*

**DOI:** 10.3390/molecules29010050

**Published:** 2023-12-20

**Authors:** Wuxia Zhang, Jiaqi He, Danping Zheng, Panpan Zhao, Yingdong Wang, Jinzhong Zhao, Peng Li

**Affiliations:** Shanxi Key Laboratory for Modernization of TCVM, Department of Basic Sciences, Shanxi Agricultural University, Jinzhong 030801, China; h13754915172@163.com (J.H.); z18835067116@163.com (D.Z.); zpp18269895462@163.com (P.Z.); 18232320334@139.com (Y.W.); zhaojz@sxau.edu.cn (J.Z.)

**Keywords:** *Poria cocos* polysaccharides, immunomodulatory, NF-κB signaling pathways, mannose receptor, MAPK signaling pathway

## Abstract

Polyporaceae is an important fungal family that has been a source of natural products with a range of pharmaceutical activities in China. In our previous study, two polysaccharides, PCWPW and PCWPS, with significant antioxidant and antidepressant activity were obtained from *Poria cocos*. In this study, we evaluated their potential molecular mechanisms in the immunomodulation of macrophages. PCWPW and PCWPS were characterized by GC–MS analysis to contain 1,3-linked Glcp. ELISA assays results demonstrated that the secretion of TNF-α was significantly enhanced by PCWPW/PCWPS. RNA-seq data demonstrated that PCWPS treatment modulated the expression of immune-related genes in macrophages, which was further confirmed by RT-qPCR assays. The activation of TNF-α secretion was found to be mannose receptor (MR) dependent and suppressed by MR inhibitor pretreatment. Moreover, the amount of TNF-α cytokine secretion in PCWPW/PCWPS-induced RAW264.7 cells was decreased when pretreated with NF-κB or MAPK signaling pathway inhibitors. Collectively, our results suggested that PCWPW and PCWPS possessed immunomodulatory activity that regulates TNF-α expression through the NF-κB/MAPK signaling pathway by binding to mannose receptors. Therefore, PCWPW and PCWPS isolated from *Poria cocos* have potential as drug candidates for immune-related disease treatment.

## 1. Introduction

Polysaccharides are biomacromolecules composed of monosaccharides derived from plants and have become an influential natural product class in recent years [1]. Studies on botanical polysaccharides demonstrate that they have a variety of bioactive properties, including antibacterial properties, antivirus properties, and antitumor properties [2]. Additionally, some researchers have confirmed that *Dendrobium huoshanense* polysaccharides have the potential to improve osteogenesis [3]. The compound polysaccharides derived from *Ganoderma lucidum* and *Polyporus umbellatus* display immune modulation and regulate intestinal flora, thereby providing protection against colitis [4]. Duan et al. extracted, characterized and administered *Poria cocos* polysaccharides (PCP) to mice by gavage, finding that PCP modulated the immune barrier by increasing IL-2, IL-4, IL-6, IL-10, TGF-β, and IFN-γ expression [5]. PCP-1C significantly reversed the inflammatory factors and proteins that increased with alcohol feeding; it shows that PCP-1C treatment reduced hepatic inflammation by inhibiting the TLR4/NF-κB signaling pathway [6]. In the ApoE^−/−^ mice model induced by a high-fat diet, PCP intervenes in atherosclerosis by inhibiting the activation of the TLR4/NF-κB pathway in the aorta, reducing inflammatory factors and blood lipid levels [7]. Therefore, botanical polysaccharides are believed to have potential therapeutic benefits.

Macrophages are stimulated through pattern recognition receptors (PRRs) on the cell membrane; PRRs include Toll-like receptor 4 (TLR4), mannose receptor (MR), scavenger receptor (SR), cluster of differentiation 14 (CD14), and Decin-1 [8]. Polysaccharides initiate downstream signaling via receptors in macrophages, increased cell proliferation, phagocytosis, oxygen species (ROS), cytokines, and nitric oxide (NO) expression [9,10]. The NF-κB and MAPK signaling pathways play a key role in mediating immune response. *Ficus carica* polysaccharides regulated macrophages via the activation of the NF-κB and MAPK signaling pathways [11]. *Ophiocordyceps sinensis* polysaccharides mediated MAPK signaling pathways to release NO and cytokines [12].

*Poria cocos* was mainly distributed along the central south and east of China. Aside from being a diuretic and spleen stimulant, it also tones the heart and calms the spirit [13]. In a previous study, we obtained two polysaccharides, PCWPW and PCWPS, from *Poria cocos*, and determined their basic chemical composition and antidepressant activity. The results showed that PCWPW and PCWPS containing mannose and fucose had good antidepressant activity, and their antidepressant mechanisms may be related to their potential immune regulatory functions [14]. Therefore, in this article, methylation analyses, immunomodulatory effects and the mechanisms of PCWPW and PCWPS were explored. The levels of inflammatory factors and several related immunological events and relative mechanisms were tested by RNA-seq, ELISA, and qRT-PCR experiments.

## 2. Results

### 2.1. Analysis of the Glycosidic Linkages of Polysaccharides

Previous research has shown that PCWPW and PCWPS were mainly composed of mannose, glucose, galactose and fucose. Methylation was a common method to investigate the intrachain glycosidic linkages between monosaccharide residues. The results of the GC–MS analysis were shown in Table 1. The main chain of PCWPW and PCWPS were 1,3-linked-Glcp with a molar ratio of 78.39% and 72.78%, containing branches of 1,4-linked-Glcp, 1,6-linked-Glcp, and 1,3,6-linked-Glcp. Meanwhile, a small number of terminal groups, 1,3,-linked Fucp, 1,5-linked Araf, 1,6-linked Galp, and 1,2,6-linked Manp residues were found in PCWPW and PCWPS. Moreover, PCWPS was found to contain 1,5-linked Araf, 1,3,6-linked Galp, and residues, while none was detected in the PCWPW.

### 2.2. Effects of PCWPW and PCWPS on the TNF-α Production and mRNA Expressions in RAW264.7 Cells

**Figure 1:**
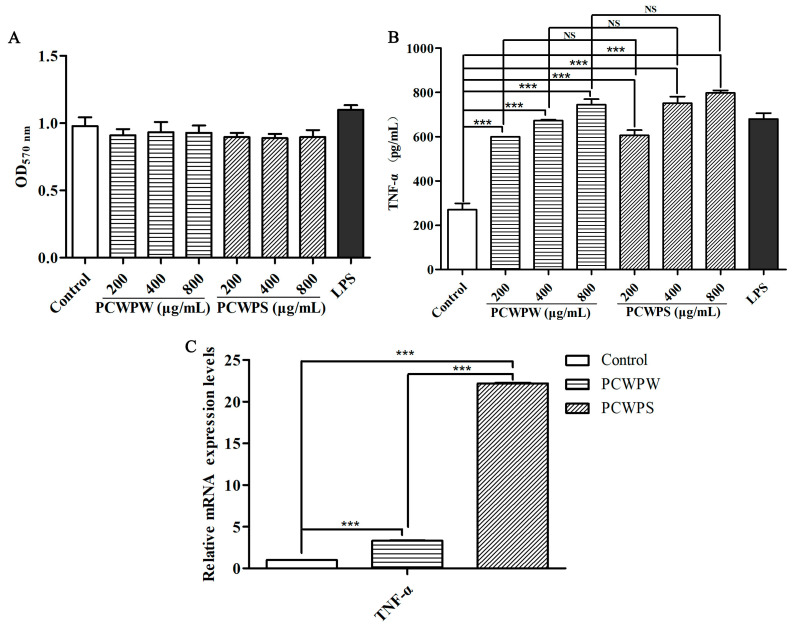
Effects of PCWPW and PCWPS on cell proliferation (**A**), TNF-α production in RAW264.7 cells (**B**), and the mRNA expression of TNF-α genes (**C**). Data were expressed as the mean ± SD, “NS” means no significant difference, (***) *p* < 0.001, ANOVA with Tukey’s HSD test.

Activated macrophages eliminated invading pathogens and damaged cells by producing various proinflammatory cytokines. As a major proinflammatory cytokine, TNF-α promoted cell proliferation and participated in systemic immunity [15]. To determine whether PCWPW and PCWPS could stimulate TNF-α production, macrophages were treated with different doses of PCWPW and PCWPS for 24 h, and TNF-α production in culture supernatants was measured by ELISA assay. As shown in Figure 1A, compared with the blank group, PCWPW and PCWPS at a concentration in the range of 200~800 μg/mL had no cytotoxicity on RAW264.7 cells. In Figure 1B, compared with the blank group, different concentrations of PCWPW and PCWPS significantly stimulated the secretions of TNF-α with a significant dose effect by RAW264.7 cells. Compared to polysaccharide ECIP-1A from *Eurotium cristatum*, when the concentration of PCWPS was 200 µg/mL, the amount of macrophage secretion was the same as that of ECIP-1A at 500 µg/mL [16]. At the transcriptional level, the qRT-PCR method was used for relative quantification to detect the transcriptional levels of cytokines, and actin-β was used as the reference gene. The mRNA levels of TNF-α were significantly increased compared with the negative control (Figure 1C). PCWPS at 800 µg/mL showed a statistically significant increase in transcription levels compared with PCWPW at 800 µg/mL, which was consistent with the results of ELISA. Experimental results revealed that PCWPW and PCWPS improved immune response by stimulating macrophages to secrete factor TNF-α.

### 2.3. PCWPW and PCWPS Activated Macrophages by Binding to Mannose Receptor (MR)

**Figure 2:**
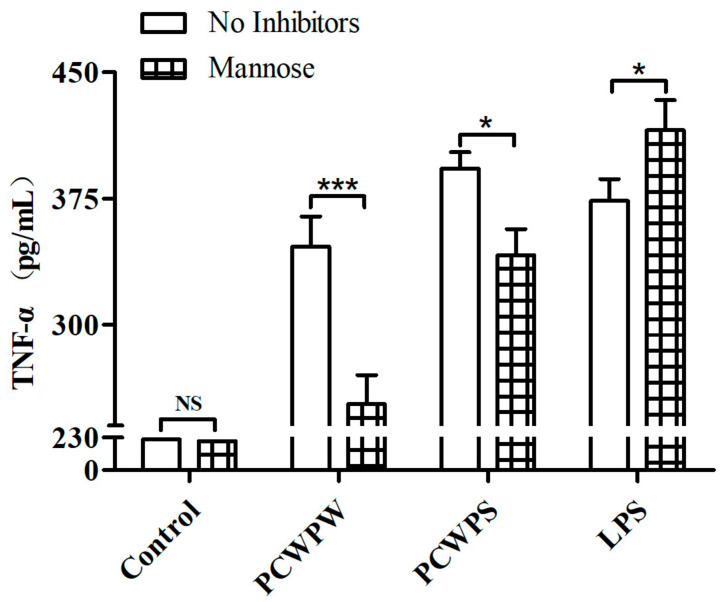
Effect of MR inhibitors on TNF-α production in RAW264.7 cells stimulated by PCWPW and PCWPS (800 μg/mL). “NS” means no significant difference, (*) *p* < 0.05 and (***) *p* < 0.001, ANOVA with Tukey’s HSD test.

A previous study reported that botanical polysaccharides recognized and bound cell surface receptors MR on RAW264.7 cells [17]. To determine whether PCWPW and PCWPS upregulated TNF-α production via binding to MR on cells, we measured secreted TNF-α by ELISA after pretreating with mannose. The ELISA results were shown in Figure 2, compared with the untreated group, the secretion of TNF-α in the mannose-treated group was significantly reduced, which declined from 346.30 ± 14.66 pg/mL and 392.74 ± 8.09 pg/mL to 252.79 ± 14.16 pg/mL and 341.34 ± 12.64 pg/mL, respectively. It suggested that the mannose-treated group effectively blocked the binding of PCWPW or PCWPS to mannose receptor in macrophages. It suggested that TNF-α-mediated inflammatory processes on RAW264.7 cells were linked to the MR.

### 2.4. MAPK Pathway Activation Was Responsible for the Release of Proinflammatory Cytokines

**Figure 3:**
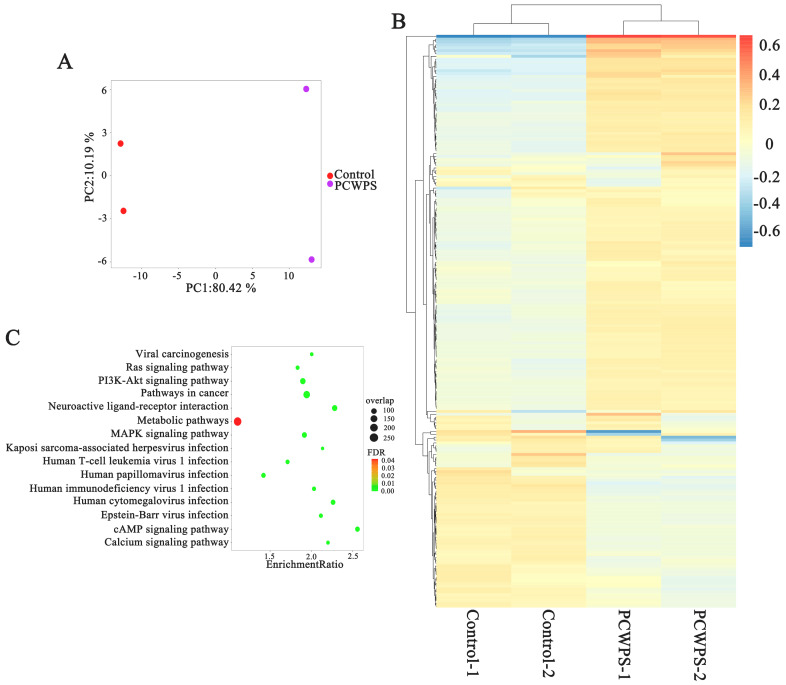
(**A**) Transcriptomic data from RNA-seq PCA analysis. (**B**) Heat maps of clusters of differentially expressed genes. (**C**) A bubble chart displaying KEGG pathway enrichment.

To investigate the molecular mechanism of macrophage activation by *Poria cocos* polysaccharides, RAW264.7 cells grown to the log phase were treated with PBS (control) and PCWPS for 12 h, respectively. Total RNA was extracted for transcriptome sequencing, and the differential genes between the control and experimental groups were analyzed. The PBS and PCWPS treatment groups showed significant differences in cluster analysis and principal component analysis (Figure 3A,B). The expression of 274 genes was significantly changed (*p* value ≤ 0.05), among which were 44 upregulated genes (log2Fold-Change (log2FC) > 1) and 14 downregulated genes (log2FC) < −1). Pathway analysis was carried out using the KEGG database on these differential genes. Figure 3C shows that the differentially expressed genes were mainly concentrated in the “MAPK signaling pathway”, the “cAMP signaling pathway”, the “calcium signaling pathway” and the “PI3K-Akt signaling pathway”.

MAPK has four subfamilies, corresponding to four MAPK pathways: ERK1/2, JNK1/2/3, p38, and BMK1 (ERK5). MAPK signaling molecules play an important role in cell growth and differentiation as well as in the induction of cytokine expression [11]. To further explore MAPK signaling pathways that may be involved in the immune activation of PCWPW and PCWPS, qRT-PCR and inhibitor experiments were used to detect whether the activation of macrophages by PCWPW and PCWPS was related to the MAPK signaling pathway.

**Figure 4:**
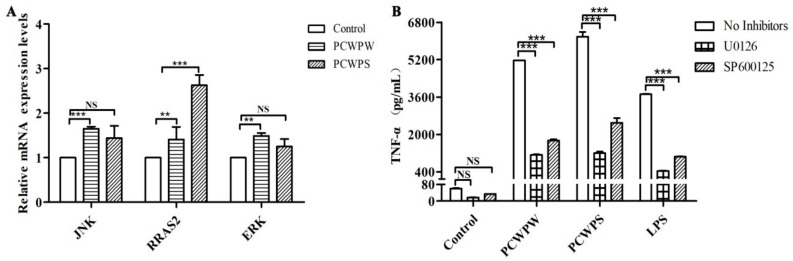
The quantitative analysis of PCWPW and PCWPS on the mRNA expression of MAPK signaling pathways in RAW264.7 cells (**A**). The effect of JNK and ERK inhibitors (U0126 and SP600125) on TNF-α production in RAW264.7 cell was measured by ELISA (**B**). Cells were treated with 800 µg/mL of PCWPW and PCWPS incubated for 24 h with the MAPK inhibitors: U0126 (10 μM) and SP600125 (20 μM). “NS” means no significant difference, (**) *p* < 0.01 and (***) *p* < 0.001 ANOVA with Tukey’s HSD test.

In vitro experiments showed that the expression levels of RRAS2 genes were significantly upregulated several-fold by PCWPW and PCWPS. In addition, PCWPW also upregulated the expression of JNK and ERK, whereas PCWPS upregulated these two genes, but not significantly (Figure 4A). In the subsequent experiments, ELISA was used to verify that PCWPW and PCWPS stimulated macrophages through the MAPK pathway. We added inhibitors U0126 (ERK1/2-MAPK inhibitor) and SP600125 (JNK1/2-MAPK inhibitor) to block the corresponding signaling pathway in RAW264.7 cells, then detected the content of cytokine TNF-α in the supernatant. According to the production instructions, the recommended concentration of U0126/SP600125 was 1–20 μM/1–100 μM, respectively. After the preliminary experiment, we found that the final concentration of U0126 (10 µM) and SP600125 (20 µM) has an ideal effect, and the inhibitory effect of this concentration on the signaling pathway is much higher than its corresponding IC_50_. As shown in Figure 4B, the inhibitors had no toxic effect on the cells in the control group, and the inhibitor treatments significantly repressed the TNF-α expression of PCWPW/PCWPS induced in RAW264.7 cells. In conclusion, PCWPW and PCWPS, through the MAPK pathway, activated the macrophages to secrete TNF-α to exert their immunomodulatory function.

### 2.5. NF-κB Mediated the Immune Activity of PCWPW and PCWPS

The MAPK signaling pathway is one of the upstream pathways of NF-κB, and phosphorylation of ERK, JNK, and p38 activates the NF-κB signaling pathway or other transcription factors [18]. NF-κB is associated with NF-κB inhibitor (IκB) binding and exists in the cytoplasm in a static state. When macrophages are activated, the IκB kinase (IKK) complex converts IκB phosphorylation, and NF-κB is released from the cytoplasm and translocated to the nucleus, leading to the release of proinflammatory cytokines [19].

**Figure 5:**
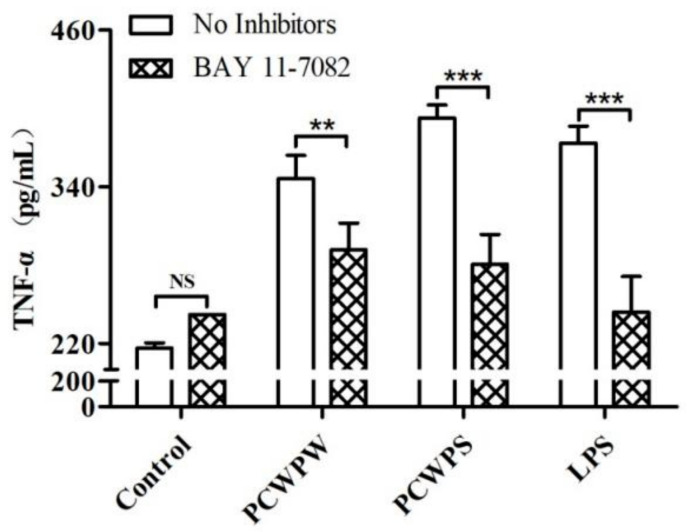
Effect of NF-κB inhibitors on TNF-α production in RAW264.7 cells stimulated by PCWPW and PCWPS (800 μg/mL). “NS” means no significant difference, (**) *p* < 0.01 and (***) *p* < 0.001, ANOVA with Tukey’s HSD test.

We used an NF-κB inhibitor to determine whether NF-κB participates in the reaction process of macrophage activation by PCWPW or PCWPS. As shown in Figure 5, the inhibitors had no toxic effect on the cells in the control group. For the PCWPW and PCWPS groups, the addition of the NF-κB specific inhibitor significantly decreased the production of TNF-α. Thus, we proved that PCWPW and PCWPS may promote the proinflammatory cytokine TNF-α production through the NF-κB signaling pathway in macrophages.

## 3. Discussion

In this study, the chemical characteristic analysis demonstrated that the main structure of PCWPW and PCWPS comprised 1,3-linked-Glcp-containing branches of 1,4-linked-Glcp, 1,6-linked-Glcp, and 1,3,6-linked-Glcp, and also contains some fucose, mannose, galactose and arabinose residues. The activity of the polysaccharide was affected by the type of the backbone and the degree of branching [20]. Polysaccharides from the *Amanita muscaria* have a (1→6)-linked Galp main chain, which selectively inhibits the proliferation of B16-F10 melanoma cells [21]. The glycyrrhiza polysaccharides with 1,4-linked-Galp main chains promoted dendritic cells maturation, antigen presentation and phagocytosis [22].

It has been shown that polysaccharides modulated the immune system by regulating immune cells [23]. Macrophage activation played an important role in immunological activity, and this activation secretes cytokines such as TNF-α, interleukin-1 (IL-1), IL-6, and interferon (IFN)-α/β participated in the regulation of cellular defense [24]. TNF-α is a representative cytokine that supports the immune system and induces inflammation. We assessed the immunomodulatory effects of PCWPW and PCWPS by measuring this cytokine secretion from cells. ELISA results showed that PCWPW and PCWPS effectively regulated proinflammatory cytokine TNF-α. According to transcriptome sequencing results, PCWPS has a regulatory effect on immune-related pathways in macrophages. At the same time, qRT-PCR showed that the expression level of TNF-α-related mRNA in PCWPW- and PCWPS-stimulated cells was significantly higher than that in the blank control group. In these results, PCWPW and PCWPS were found to have an impact on immune activation by macrophages. As the molecular weight of the polysaccharides was higher, more repeating structures bind to receptors or membrane targets, and therefore performed considerable bioactivities [25]. Polysaccharides were capable of binding to PRRs on the surface of macrophages which activated several intracellular signaling pathways [26]. PCWPW and PCWPS with mannose and fucose residues bind to MR, exerting immunomodulatory effects by activating macrophages. Additionally, The molecular weight of polysaccharides is an important factor affecting the biological activity of polysaccharides [27]. PCWPS induced relatively stronger activation in macrophages (the molecular weight of PCWPW is 37,154 Da, and that of PCWPS is 186,209 Da). Among the three kinds of *Astragali Radix* polysaccharides with different molecular weights obtained by using ultrafiltration, the one with a larger molecular weight had the strongest immune-enhancing activity [28].

MAPK and NF-κB regulate the immunity systems, cell growth, and differentiation by releasing the expression of various proinflammatory genes and proteins [29]. *Apocynum venetum* L. flower polysaccharides activate the MAPK and NF-κB pathways to enhance the immune function of RAW264.7 cells [30]. It has also been reported that mung bean skin polysaccharide exerts an immunomodulatory effect on RAW264.7 cells through TLR4-mediated MAPKs and NF-κB signaling pathways [31]. The immunomodulatory effect of *Isaria cicadae Miquel* polysaccharide was related to the TLR4-MAPK-NF-κB pathway, which performed an immunomodulatory effect by promoting NO, TNF-α, and IL-6 secretion [32]. Polysaccharides extracted from *Taraxacum platycarpum* root bind to TLR4, TLR2, and CR3 receptors on macrophages, activate the MAPK and NF-κB pathways, upregulate mRNA expression and produce related cytokines [33]. To further explore the potential mechanism of macrophage activation, the corresponding signaling pathway inhibitors were used to block the downstream signal pathway in this study. In contrast to the polysaccharide group, if the addition of the inhibitors reduced the level of TNF-α secretion, this would indicate that the polysaccharide exerted an immunomodulatory effect through this signaling pathway. Results obtained from inhibitor studies further demonstrate that PCAPW and PCAPS play an immune role through the MAPK and NF-κB pathways. Hence, these demonstrated that PCWPW and PCWPS obtained from the *Poria cocos* could interact with receptor MR on the surface of macrophages, increasing TNF-α secretion and mRNA expression in cells via the activation of the NF-κB and MAPK pathways. It is recorded in the Compendium of Materia Medica that “Fuling is beneficial to spleen and stomach and tonifying lung deficiency”. The following is also recorded in Bencao Huiyan: “*Poria* replenishing the spleen and benefiting the lung, benefiting water and wetting, calming the mind and benefiting the kidney” [34]. *Poria cocos* is used as an active ingredient of over-the-counter health supplements and medicine in traditional medicine, such as “Bu-Zhong-Yi-QiTang” used for treating gastrointestinal diseases, allergic rhinitis, and atopic dermatitis [35]. Studies have also shown that inflammation leads to spleen deficiency and illnesses of the stomach and intestines [36]. TNF-α is a proinflammatory cytokine involved in normal inflammatory and immune responses, which coordinates the production of other cytokines, cell survival and death to coordinate tissue homeostasis. In this study, PCWPW and PCWPS obtained from the *Poria cocos* interact with receptor MR on the surface of macrophages, increasing TNF-α secretion and mRNA expression in cells via the activation of the NF-κB and MAPK pathways, which indicates that it may be effective in these diseases.

## 4. Materials and Methods

### 4.1. Materials and Chemicals

As previously mentioned, PCWPW (37,154 Da) and PCWPS (186,209 Da) were extracted using a water-extraction alcohol-precipitation method and purified by DEAE-52 cellulose chromatography [13]. Lipopolysaccharide (LPS) was purchased from Sigma-Aldrich (St. Louis, MO, USA). (*E*)-3-[(4-methylphenylsulfonyl]-2-propionitrile (BAY 11-7082, NF-κB inhibitor) was purchased from Beyotime Biotechnology (Shanghai, China). U0126 (MEK1/2 inhibitor) and SP600125 (JNK1/2/3 inhibitor) were purchased from MedChemExpress (Shanghai, China). ELISA kits were obtained from R&D Systems (Minneapolis, MN, USA). All other chemicals used were of analytical grade.

### 4.2. Methylation Analysis

The methylation analysis was performed based on Oladele et al. [37]. Samples were dissolved in DMSO and then added to NaOH. Iodomethane was added for methylation and permitted to react, excess potassium iodide was extracted and lyophilized by dialysis. They were performed on a GC–MS system (Shimadzu GCMS-QP 2010, Shimadzu, Japan) with an RXI-5 SIL MS (30 m × 0.25 mm × 0.25 μm, SHIMADZU, Shanghai, China) column. The column temperature was raised from 120 °C to 250 °C at a rate of 3 °C/min. Helium was used as the carrier gas and maintained at a flow rate of 1 mL/min.

### 4.3. Determine the Secretion of TNF-α Induced by PCWPW and PCWPS

The RAW264.7 cells line was obtained from Prof. Jinyou Duan, Northwest A&F University. Cells were treated with a series of concentrations of PCWPW or PCWPS (200, 400, and 800 μg/mL) and incubated for 24 h. The equivalent volumes of LPS (2.5 μg/mL) and phosphate-buffered saline (PBS) were used as a positive control and a blank control, respectively. The culture supernatants were collected, and the contents of TNF-α were assayed by ELISA kits according to the instructions [38].

### 4.4. Investigation of Mannose Receptor

Macrophages were characterized by their surface expression of mannose receptor (MR), which can recognize mannose, fucose, and N-acetyl-glucosamine, and activates the NF-κB signaling pathway, playing an essential role in immune homeostasis [39]. Monosaccharide composition results showed that PCWPW and PCWPS contain mannose and fucose residues. To determine whether MR was relevant to PCWPW- or PCWPS-induced cytokine production, RAW264.7 cells were pretreated with mannose (500 µg/mL) for 1 h in the presence of 800 μg/mL PCWPW or PCWPS for 24 h, then the TNF-α content was measured by using ELISA kits.

To further confirm the possible mechanism involved in PCWPW/PCWPS-mediated activation in cytokine expression, NF-κB and MAPK inhibitors, BAY 11-7082 (2 µM), U0126 (10 μM), and SP600125 (20 μM), were incubated with cells. Cytokine contents in supernatants were determined by the ELISA kits with PBS or inhibitors for 1 h before incubation with 800 μg/mL PCWPW or PCWPS for 24 h.

### 4.5. Extraction, Preparation, and Sequencing of RNA-Seq Libraries

Briefly, RAW264.7 cells (5 × 10^6^/well) were grown in 6-well plates in RPMI-1640 medium, containing 10% fetal bovine serum (FBS) incubated at 37 °C under an atmosphere of 5% CO_2_ for 12 h. An Illumina HiSeqTM 4000 platform (Majorbio Bio-Pharm Technology Co., Ltd., Shanghai, China) was used to sequence the total RNA extracted from the cells. Salmon 1.10.2 software was initially used to count transcripts, and tximport was used to process Salmon’s estimated read counts per transcript [40]. The rlog function from the DESeq2 (version: 1.28.1) package was used to transform the read counts from the RNA-seq alignment, and the DEseq2 method was used to identify genes with differential expression [41].

### 4.6. Target Prediction Based on Differential Gene Expression Profiles

Potential targets of PCWPS were predicted using the transcriptome-based multi-scale network pharmacological platform (TMNP: http://www.bcxnfz.top/TMNP/, accessed on 18 January 2023) [42]. Gene ontology (GO, http://geneontology.org/, accessed on 18 January 2023) was used for enrichment analysis of GO pathways and the Kyoto Encyclopedia of Genes and Genomes (KEGG, https://www.kegg.jp/, accessed on 18 January 2023) was used for enrichment analysis of KEGG pathways. Our analysis focused on GO terms and KEGG pathways with a *p* value (FDR) less than 0.05 [43].

### 4.7. Analysis of Transcription Levels of Differentially Expressed Genes

In order to validate RNA-seq results, RT-qPCR was performed on differentially expressed genes. RAW264.7 cells were treated with 800 µg/mL PCWPW or PCWPS for 6 h. The cells were collected for RNA extraction using a Total RNA Kit I (OMEGA, Norcross, GA, USA) And reversed to cDNA by using a PrimeScriptTMRT Master Mix reagent kit (TaKaRa, Shiga, Japan) according to the instructions. The expression of mRNA was performed with a TB Green^®^ Premix Ex TaqTM II (TaKaRa, Shiga, Japan) on a QuantStudio 6 (ABI, Foster City, CA, USA) detection system, and its reaction conditions were 95 °C for 30 s, 95 °C denaturation for 5 s, 60 °C annealing for 30 s, and 40 cycles. The primer sequences are as listed in Table 2. The expression levels of mRNA were calculated by using the 2^−ΔΔCt^ method, and mRNA abundances were normalized by actin-β [44].

### 4.8. Cellula Pathway Inhibitor Assay

The inhibitor of extracellular signal-regulated kinases (ERK1/2), c-Jun NH2-terminal kinases (JNK1/2/3) and the NF-κB pathway were added into experiment to analyze the roles of these pathways in RAW264.7 macrophage immunity. Cells were preincubated with specific pathways inhibitors: the ERK1/2 pathway inhibitor, U0126 (10 μM), the JNK1/2/3 inhibitor, SP600125 (20 μM), and the NF-κB pathway inhibitor, BAY 11-7082 (2 µM) for 1 h. Following 24 h of culture, the supernatants were collected for TNF-α quantification.

### 4.9. Statistical Analysis

Data were repeated in at least three independent experiments for each sample, analyzed statistically by GraphPad Prism 9.0, and expressed as the mean ± SD. Statistical significance was calculated by ANOVA analysis with Tukey’s HSD test.

## 5. Conclusions

PCWPW and PCWPS from the *Poria cocos* had a backbone composed of 1,3-Glcp, and also contain some fucose and mannose residues, which could interact with mannose receptor on the surface of macrophages, increasing TNF-α secretion and mRNA expression in cells. The inhibitor studies further demonstrate that PCAPW and PCAPS activate immune response through the MAPK and NF-κB pathways. The two polysaccharides, PCWPW and PCWPS, from *Poria cocos* had the potential for immunoregulatory activity, which could be used for the development of drugs to improve immunity or as immune adjuvants.

## Figures and Tables

**Table 1:** Methylation analysis of PCWPW and PCWPS.

Type of Linkage	Mass Fragments (*m*/*z*)	Molar Ratios (%)	
		PCWPW	PCWPS
Araf-1	43, 71, 87, 101, 117, 129, 145, 161	-	0.2
Fucp-1-3	43, 89, 101, 117, 131, 159, 173, 233	0.4	0.85
Glcp-1	43, 71, 87, 101, 117, 129, 145, 161, 205	5.07	3.99
Araf-1-5	43, 71, 87, 99, 101, 117, 129, 161, 189	0.78	1.85
Glcp-1-3	43, 71, 87, 99, 101, 117, 129, 161, 173, 233	78.39	72.78
Glcp-1-4	43, 71, 87, 99, 101, 113, 117, 129, 131, 161, 173, 233	3.24	1.49
Glcp-1-6	43, 71, 87, 99, 101, 117, 129, 161, 173, 189, 233	3.67	2.38
Galp-1-6	43, 71, 87, 99, 101, 117, 129, 161, 173, 189, 233	1.81	4.61
Glcp-1-3-6	43, 87, 99, 101, 117, 129, 139, 159, 173, 189, 233	5.57	4.72
Manp-1-2-6	43, 87, 99, 113, 129, 173, 189, 233	1.07	3.65
Manp-1-2	43,87,129,161,189	-	1.97
Galp-1-3-6	43,87,117,129,159,189,233	-	1.51

**Table 2:** The primer sequences in this study.

Primer Name	Sequences
actin-β	Forward: 5′-TCACCCACACTGTGCCCATCTACGA-3′
	Reverse: 5′-GGATGCCACAGGATTCCATACCCA-3′
TNF-α	Forward: 5′-ATAGCTCCCAGAAAAGCAAGC-3′
	Reverse: 5′-CACCCCGAAGTTCAGTAGACA-3′
JNK	Forward: 5′-ATTGAACAGCTCGGAACACC-3′
	Reverse: 5′-GAGTCAGCTGGGAAAAGCAC-3′
RRAS2	Forward: 5′-AATGCCCTCCTTCACCAG-3′
	Reverse: 5′-GCAGCCTTTCTTGTCTTTT-3′
ERK	Forward: 5′-TGACCTCAAGCCTTCCAACC-3′
	Reverse: 5′-ATCTGGATCTGCAACACGGG-3′

## Data Availability

Data are contained within the article.
